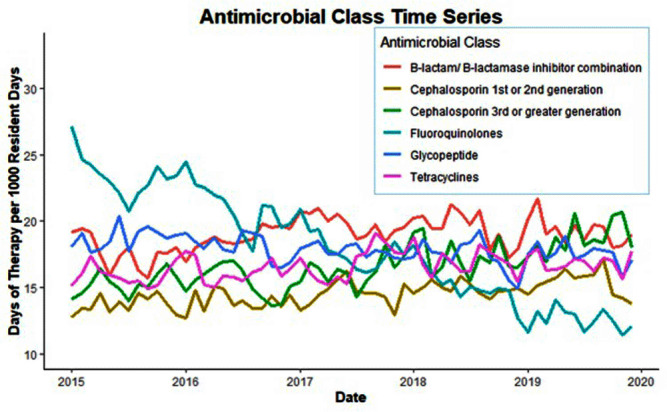# Antimicrobial Use in Veterans Affairs Community Living Centers, 2015 - 2019

**DOI:** 10.1017/ash.2024.102

**Published:** 2024-09-16

**Authors:** Christian Dalton, Tina Willson, Brigid Wilson, Taissa Bej, Nadim El Chakhtoura, Sunah Song, Oteshia Hicks, Corinne Kowal, Makoto Jones, Steven Handler, Robin Jump, Vanessa Stevens

**Affiliations:** University of Utah; University of Utah Division of Epidemiology; Northeast Ohio VA Healthcare System; Institute for Computational Biology; Louis Stokes Cleveland VA Medical Center; Department of Veteran Affairs; VA Pittsburgh Healthcare System; US Dept Veterans Affairs

## Abstract

**Background:** Optimizing antimicrobial use (AU) among post-acute and long-term care (PALTC) residents is fundamental to reducing the morbidity and mortality associated with multidrug-resistant organism (MDROs), as well as unintended social consequences related to infection prevention. Data on AU in PALTC settings remains limited. The U.S. Department of Veteran Affairs (VA) provides PALTC to over 23,000 residents at 134 community living centers (CLCs) across the United States annually. Here, we describe AU in VA CLCs, assessing both class and length of therapy. **Methods:** Monthly AU between January 1, 2015 and December 31, 2019 was extracted from the VA Corporate Data Warehouse across 134 VA CLCs. Antimicrobials and administration routes were based on the National Healthcare Safety Network AU Option protocol for hospitals. Rates of AU were measured as the days of therapy (DOT) per 1,000 resident-days. An antimicrobial course was defined as the same drug and route administered to the same resident with a gap of ≤ three days between administrations. Course duration was measured in days. AU Rates were measured as the days of therapy (DOT) per 1,000 resident-days. **Results:** The most common class of antimicrobial course administered during the study period was beta-lactam/beta-lactamase inhibitor combinations (15%) followed by fluroquinolones (14%), extended-spectrum cephalosporins (12%) and glycopeptides (11%; Figure 1). Neuraminidase inhibitors had the longest median (IQR) course duration (10 (IQR 8) days), followed by tetracyclines (8 (IQR 8) days), and then folate pathway inhibitors, nitrofurans and 1st/2nd generation cephalosporins (7 (IQR 7) days). Overall, 60% of antimicrobial courses were administered orally, with fluroquinolones the most frequently administered orally (20%). From 2015 – 2019, the annual rate of total antimicrobial use across VA CLCs decreased slightly from 213.6 to 202.5 DOT/1,000 resident-days. During the 5-year study period, fluroquinolone use decreased from 27.47 to 13.36 DOTs/1,000 resident-days. First and 2nd generation cephalosporin use remained relatively stable, but 3rd or greater generation cephalosporin use increased from 14.70 to 19.21 DOTs/1,000 resident-days (Figure 2). **Conclusion:** The marked decrease in the use of fluoroquinolones at VA CLCs from 2015-2019 is similar to patterns observed for VA hospitals and for non-VA PALTC facilities. The overall use of antibacterial agents at VA CLCs decreased slightly during the study period, but other broad-spectrum agents such as 3rd or greater generation cephalosporins increased over the same period. The strategies used to decrease fluroquinolone use may have application for other antibiotic classes, both in VA and non-VA PALTC settings.

**Disclosure:** Robin Jump: Research support to my institution from Merck and Pfizer; Advisory boards for Pfizer

**Figure:**
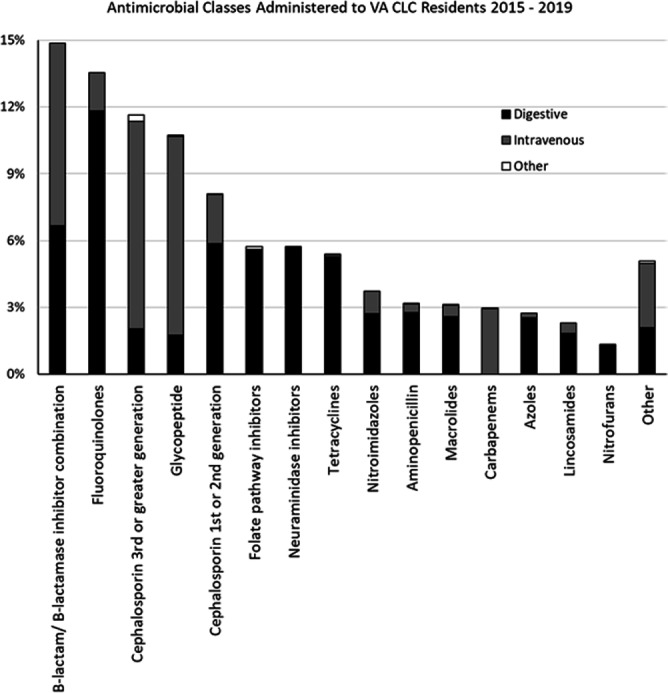

**Figure:**